# The importance of examining an active immune system during immunotherapy

**DOI:** 10.18632/oncotarget.26580

**Published:** 2019-01-15

**Authors:** Heather M. Gibson, Nerissa T. Viola

**Affiliations:** Heather M. Gibson: Department of Oncology, Karmanos Cancer Institute, Detroit, MI, USA; Nerissa T. Viola: Department of Oncology, Karmanos Cancer Institute, Detroit, MI, USA

**Keywords:** interferon-γ, PET, immunotherapy

Immuno-oncology has made significant strides over the past several years with a number of immunotherapy (ITx) modalities showing promise in persuading the host immune system to recognize and eradicate tumors. The present clinical practice for monitoring treatment outcomes utilizes radiographic techniques guided by RECIST 1.1 for measuring response criteria in patients. However, these guidelines were established within the framework of chemotherapeutics, where tumor volume generally depicts response. With ITx drugs, a fraction of patients may experience what falsely appears to be tumor growth, termed pseudoprogression, due to an influx of tumor-fighting immune cells. Though this is reported to occur in less than 10% of cases across tumor types [1], when one considers that overall response rate is generally less than 30%, psuedoprogression may play a significant role in misguiding downstream clinical decisions under the purview of RECIST 1.1. As such, there remains an urgent clinical need to develop predictive markers of ITx.

The main challenge lies in the lack of reliable tools for thorough evaluation of not only the presence, but also the functional status of tumor infiltrating lymphocytes (TILs). Outside of tumor volume measurement, methods of ITx evaluation include biopsy, which may have limited feasibility and only represents a small portion of a heterogeneous tumor, and thus far non-standardized peripheral immune analysis, which may not fully reflect dynamic conditions within the tumor bed. Imaging may be able to bridge these gaps, providing real-time comprehensive analysis *in situ*. In general, imaging strategies have focused on detecting TIL cell-surface markers including CD3 for total T cells, or more specifically CD8 for cytotoxic T lymphocytes (CTL), a key mediator of tumor clearance. These imaging probes have the advantage of identifying and quantifying infiltrates, but are unable to discern activated *versus* anergic/dysfunctional status.

From our perspective, we rationalize that the presence of CTL alone is an equivocal measure of successful ITx, and focus should instead be directed toward assessing activation status. A radiotracer to OX40, a cell surface antigen expressed after activation, was able to predict response to *in situ* vaccination [2]. Alternatively, a peptide-based tracer to granzyme B, which is produced with IFN-γ and perforin during CTL activation, has been shown to identify response to checkpoint blockade [3]. Others have designed tracers against checkpoint proteins, such as PD-1, PD-L1 [4, 5] and CTLA-4 [6], which may help identify patients most likely to respond to therapies targeting these pathways, however many of these molecules are activated upon immune stimulation and do not necessarily reflect whether T cells are functional or remain suppressed.

Our approach is to target interferon-γ (IFN-γ) with a radiolabeled antibody and image by positron emission tomography (PET) to identify ongoing anti-tumor activity. In our recently published paper [7] we show that IFN-γ PET can be used to monitor *in situ* response in a mouse model of HER2 cancer vaccination. IFN-γ is produced by activated CD8+ CTL, Th1-skewed CD4, and NK cells, and is a hallmark of anti-tumor immunity [8]. Despite the soluble nature of IFN-γ, we are able to visualize localized expression within the tumor bed. *In vitro* analysis suggests the antibody tracer can detect IFN-γ complexed with its receptor on tumor cells, likely contributing to tracer sequestration *in vivo*. We have demonstrated that IFN-γ PET identifies activity of intratumoral T cells and predicts therapeutic outcome after HER2 vaccination. Perhaps of greater significance is the fact that we have also tested IFN-γ PET in a model of induced T cell anergy and find low to background levels of tracer accumulation, despite the presence of infiltrating CTLs. In this system, intratumoral CTLs were largely PD-1+, suggesting they may have become anergic or exhausted. Taken together, these results justify IFN-γ PET as a potentially superior imaging modality compared to general T cell surface markers. Our imaging approach may enhance understanding of the interplay of intratumoral immune signatures before, during, and after ITx. IFN-γ PET allows unbiased visualization of dynamic host immune activity within the tumor microenvironment for prediction of efficacy, as well as to guide timely application of secondary intervention. As imaging technologies advance, it may prove beneficial to monitor multiple targets either simultaneously *via* a multi-modal approach, or in sequence using short-lived radionuclides such as ^18^F. Whole-body imaging of immune activity has great potential as a practical, non-invasive evaluation tool for tumor immunotherapy, as well as inflammatory conditions including autoimmunity and infection.
